# TBI related death has become the new epidemic in polytrauma: a 10-year prospective cohort analysis in severely injured patients

**DOI:** 10.1007/s00068-024-02653-1

**Published:** 2024-09-17

**Authors:** Karlijn J. P. van Wessem, Kim E. M. Benders, Luke P. H. Leenen, Falco Hietbrink

**Affiliations:** Department of Trauma Surgery, University Medical Center Utrecht, Suite G04.232, Heidelberglaan 100, 3584 CX Utrecht, The Netherlands

**Keywords:** Polytrauma, TBI, Mortality, Cause of death

## Abstract

**Introduction:**

Advances in trauma care have attributed to a decrease in mortality and change in cause of death. Consequently, exsanguination and traumatic brain injury (TBI) have become the most common causes of death. Exsanguination decreased by early hemorrhage control strategies, whereas TBI has become a global health problem. The aim of this study was to investigate trends in injury severity,physiology, treatment and mortality in the last decade.

**Methods:**

In 2014, a prospective cohort study was started including consecutive severely injured trauma patients > 15 years admitted to a Level-1 Trauma Center ICU. Demographics, physiology, resuscitation, and outcome parameters were prospectively collected.

**Results:**

Five hundred and seventy-eight severely injured patients with predominantly blunt injuries (94%) were included. Seventy-two percent were male with a median age of 46 (28–61) years, and ISS of 29 (22–38). Overall mortality rate was 18% (106/578) with TBI (66%, 70/106) being the largest cause of death. Less than 1% (5/578) died of exsanguination. Trend analysis of the 10-year period revealed similar mortality rates despite an ISS increase in the last 2 years. No significant differences in demographics,and physiology in ED were noted. Resuscitation strategy changed to less crystalloids and more FFP. Risk factors for mortality were age, brain injury severity, base deficit, hypoxia, and crystalloid resuscitation.

**Discussion:**

TBI was the single largest cause of death in severely injured patients in the last decade. With an aging population TBI will increase and become the next epidemic in trauma. Future research should focus on brain injury prevention and decreasing the inflammatory response in brain tissue causing secondary damage, as was previously done in other parts of the body.

**Supplementary Information:**

The online version contains supplementary material available at 10.1007/s00068-024-02653-1.

## Introduction

Improvements in both pre-hospital and in-hospital trauma care have contributed to a reduction in trauma-related mortality over the past four decades [1–3]. Analysis of cause-specific mortality have shown that this reduction was mainly attributable to a decrease in inflammatory related complications such as Multiple Organ Dysfunction Syndrome (MODS) and Adult Respiratory Distress Syndrome (ARDS) [4, 5]. As a consequence, hemorrhage and traumatic brain injury (TBI) have become the most common causes of death [2, 5, 6]. Introduction to damage control surgery, hemostatic resuscitation, and angioembolization have attributed to early control of hemorrhage. This has decreased the incidence of exsanguination related death, although several studies still report reasonably large numbers of hemorrhage related death in severely injured patients, especially in locations with high penetrating injury rates [2, 7–10].

Early diagnosis and control of hemorrhage has not only decreased death by exsanguination, but probably also influenced the severity of secondary brain damage. Further, changes in the epidemiology of TBI in the last decade have been observed with a shift from high energy injuries in the young to low energy falls in elderly which has become the most common cause of TBI related death nowadays [2, 11, 12].

Data including our own research have shown a decrease in deaths due to exsanguination and an increase in deaths due to TBI in all trauma patients in the early years of this century [2, 3, 5, 8, 10, 11, 13]. In order to continuously monitor treatment and outcome performance of severely injured patients in our institution, a prospective population-based cohort study was started in 2014 collecting detailed data on a daily basis on demographics, physiology, treatment and outcome in severely injured patients. The aim of this study was to investigate trends in injury severity, physiology, treatment and outcome in the past decade. Further, the role of TBI as cause of death was studied over time. Additionally, it was examined whether any risk factors for mortality (besides severity of the brain injury) could be identified, that could be influenced to ameliorate outcome.

## Materials and methods

### Study setting

The research took place at an urban Level-1 trauma center. Starting in 2014, a decade-long prospective study was initiated, encompassing all trauma patients admitted consecutively to the Intensive Care Unit (ICU) at the University Medical Center Utrecht. The hospital and its service area have been described in detail previously [14]. Since July 2013, our Level-1 trauma center has maintained a dual trauma surgeon on-call system, providing round-the-clock availability. An in-house trauma surgeon is always present upon a patient's arrival in the Emergency Department (ED), with a second surgeon on-call to assist with surgeries, lead resuscitation if a new patient arrives, or handle multiple simultaneous arrivals. An experienced trauma surgeon supervises all resuscitations and decisions, even in cases with multiple patients, allowing two surgeons to perform urgent surgeries on complex injuries [15]. The study included consecutive severely injured patients admitted to the ICU directly from the ED or after urgent surgery. Exclusions were patients under 15 years, those with isolated brain injuries (Abbreviated Injury Score (AIS) head 3 or more and AIS 2 or less in other areas), asphyxiation, drowning, and burns, due to different physiological responses to severe trauma and distinct mortality and morbidity profiles [16, 17]. A flowchart detailing patient inclusion is shown in supplemental figure S1.

### Data collection

Data were collected prospectively upon arrival in the ED and daily in the ICU by the authors (KW, LL). This included patient demographics, Injury Severity Score (ISS), physiology and resuscitation parameters. The volume of crystalloid resuscitation and the use of blood products (Packed Red Blood Cells (PRBC), Fresh Frozen Plasma (FFP), and Platelets (PLT)) were recorded within the first 24 h after admission. From 2019, data on prehospital Glasgow Coma Scale (GCS) were prospectively collected. It was decided to use the first GCS on scene in order to avoid the influence of possible prehospital intubation on GCS. Denver Multiple Organ Failure (MOF) scores [18] and ARDS Berlin criteria [19] were recorded daily for up to 28 days or until ICU discharge. The Denver MOF score was preferred over other scoring systems like Marshall MODS or Sequential Organ Failure Assessment (SOFA) to avoid complications with including the Glasgow Coma Scale (GCS) in the organ failure score, as obtaining GCS can be challenging in sedated and intubated ICU trauma patients, potentially affecting the CNS organ failure score [17].

All trauma-related deaths were reviewed weekly by the trauma surgery team using a standardized format. Charts were examined, including a checklist addressing specific issues. The review form covered resource utilization, critical time intervals, cause of death, and assessments of preventability and care quality. Preventability was determined based on criteria similar to those of the American College of Surgeons [20].

The primary outcome was the trend in in-hospital mortality over the ten-year period. Additional analyses identified mortality risk factors. Other outcomes included the incidence of ARDS and MODS, ventilator days, ICU length of stay (ICU_LOS), and hospital length of stay (H_LOS).

### Ethical approval

This prospective observational registry was approved by the local ethics committee (reference number WAG/mb/16/026664).

### Statistical analysis

Data were analyzed using IBM SPSS Statistics, version 29.0 (Armonk, NY, USA). Graphs were prepared with GraphPad Prism version 10.3.0 (San Diego, CA, USA). Results are presented as median and interquartile range (IQR). Comparison of variables was done using Kruskal–Wallis test or Pearson-Chi-square test in dichotomous data. Variables with univariate statistical significance of less than 0.10 were included in a multivariate logistic regression analysis. These variables were analyzed with forward stepwise selection to identify independent risk factors for mortality and presented as odds ratios and 95% confidence intervals. Statistical significance was defined as P < 0.05.

## Results

During the 10-year study period 578 severely multiple injured patients admitted to ICU were included. Seventy-two percent were male with a median age of 46 (28–61) years. Ninety-four percent of patients sustained a blunt injury with a median ISS of 29 (22–38). Most severe injuries were in the head and chest region; sixty percent of patients had an AIS_head ≥ 3. The different types of traumatic brain injury are described in supplemental Table S1. Seventy-one patients had an AIS_chest ≥ 3. Sixty-five percent of patients (n = 378) underwent urgent surgery (≤ 24 h), 25% of patients (n = 145) had a laparotomy, and 9% (n = 33) underwent a decompressive craniotomy. Median prehospital time (time from emergency call to dispatch of the ambulance service to arrival in ED) was 1:00 h (0:56–1:09). First measured systolic blood pressure (SBP) in ED was 120 (95–140) mmHg, initial hemoglobin (Hb) 8.2 (7.3–8.9) mmol/l, and initial base deficit (BD) in ED -3.0 (-7.0–0.0) mEq/L. Patients received 4.3 (2.3–6.0) L of crystalloids ≤ 8 h and 7.0 (4.6–9.6) L ≤ 24 h, a total of 3 units of blood products ≤ 8 h, and 4 units ≤ 24 h (2 (0–5) units of PRBC ≤ 8 h and 2 (0–6) units ≤ 24 h, 1 (0–5) unit of FFP ≤ 8 h and 2 (0–6) units ≤ 24 h, and 0 (0–1)units of PLT ≤ 8 h and 0 (0–1) ≤ 24 h). GCS was registered in 287 patients (50%) with a median GCS of 12 (6–15); Forty-nine percent of patients had a GCS of 13–15, 13% a GCS of 9–12 and 38% had a GCS of 8 or lower. Ninety-four (16%) patients developed MODS based on the Denver Score, and 17 (3%) patients developed ARDS. Patients were on the ventilator for 5 (2–10) days, 7 (3–13) days in ICU, and 20 (11–32) days in hospital.

A total of 106 patients (18%) died with TBI as the most common cause of death, followed by respiratory insufficiency (7.5%). Five patients (4.7%) died of exsanguination, all within 24 h after arrival. During the studied decade, 12% of patients (70/578) died due to TBI, 1, 2% (8/578) due to respiratory insufficiency, and 0.9% (5/578) due to hemorrhage. All other causes of death (including MODS, ARDS, sepsis, ischemia, hypoxia, high cervical spinal injury, and cardiac origin) occurred in ≤ 1% of studied population.

Median time to death was 6 (2–21) days. Thirty-one patients (29%) died within 24 h after trauma (20 due to TBI, 5 due to hemorrhage, 3 due to high cervical spinal injury, 1 due to ischemia, 1 due to cardiac injury, and in 1 patient treatment was withdrawn because of the combination of injury severity and old age), 10 of them went directly to OR from ED.

After reviewing all deaths it was concluded that death by hemorrhage could possibly be prevented in one patient (with severe chest and abdominal injuries) if the patient was immediately transported from ED to OR instead of CT prior to OR.

Multivariate analysis showed that age, AIS_head, base deficit in ED (BD_ED), BD_ICU, partial oxygen pressure in ICU (PaO2_ICU), and crystalloids ≤ 24 h were independent predictors for mortality in the studied population (Table 1).

**Table 1:** Multivariate analysis: mortality

Variable	β coefficient	P-value	Odds ratio	95% C.I.	
				Lower	Upper
Age	0.055	< 0.001	1.057	1.039	1.075
AIS_head	0.626	< 0.001	1.871	1.510	2.319
BD_ED	− 0.009	0.004	0.991	0.985	0.997
BD_ICU	− 0.012	0.008	0.988	0.979	0.997
PaO2_ICU	− 0.007	0.017	0.993	0.987	0.999
Crystalloids ≤ 24 h	0.000	0.005	1.000	1.000	1.000
Constant	− 7.455	< 0.001	0.001		

*C.I.* confidence interval, *AIS* abbreviated injury scale, *BD* base deficit, *ED* emergency department, *ICU* intensive care unit, *PaO2* partial pressure of oxygen

### Trends in time

#### Demographics and physiology

**Fig. 1:**
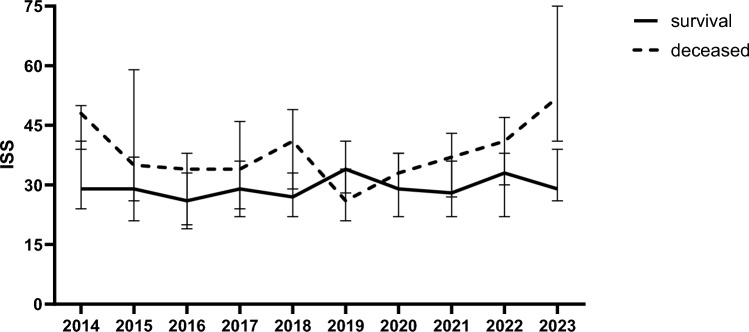
Injury Severity Score (ISS) related to mortality during the studied period. Data are expressed in median(IQR)

Age, gender, and mechanism of injury did not significantly change over the 10-year time period. There was also no difference in prehospital intubation, nor in number of patients who needed urgent surgery. Over the years, there was an increase in pelvic fractures and an increase in ISS in the last 2 years (Table 2). This was mainly attributed by the increase in ISS in patients who later died (p = 0.009, Fig. 1). Physiology on arrival in ED did not change over the years, except for prothrombin time (PT) measured in ED which was longer in the first years compared to later years (P < 0.001). Over the years first measured hemoglobin (Hb) in ICU increased, whereas base deficit (BD) and bicarbonate (HCO3^−^) in ICU decreased despite similar pH (Table 2).

**Table 2:** demographics and physiology per studied year

	2014 (n = 45)	2015 (n = 49)	2016 (n = 64)	2017 (n = 64)	2018 (n = 64)	2019 (n = 68)	2020 (n = 68)	2021 (n = 64)	2022 (n = 50)	2023 (n = 42)	P-value
Age (years)	35 (23–56)	39 (26–57)	49 (32–68)	53 (33–61)	45 (26–59)	51 (31–70)	37 (27–58)	47 (31–61)	39 (29–61)	48 (31–59)	0.09
Male gender (%)	34 (79)	38 (83)	42 (68)	44 (79)	41 (68)	37 (57)	45 (69)	43 (70)	37 (77)	32 (84)	0.08
Blunt MOI (%)	43 (95)	48 (98)	62 (97)	59 (95)	63 (98)	64 (94)	63 (92)	55 (85)	45 (90)	40 (95)	0.13
Glasgow Coma Scale						12 (7–15)	12 (6–15)	14 (8–15)	9 (3–15)	13 (4–15)	0.78
Prehospital intubation (%)	19 (42)	25 (51)	33 (52)	30 (47)	38 (59)	33 (49)	33 (49)	26 (41)	16 (32)	19 (45)	0.72
Pelvic fracture (%)	9 (19)	15 (28)	18 (27)	25 (41)	24(37)	20 (28)	16 (23)	15 (25)	20 (42)	22 (50)	0.02*
Laparotomy (%)	10 (22)	15 (28)	8 (13)	24 (38)	17 (27)	18 (26)	12 (18)	22 (34)	9 (18)	10 (24)	0.03*
Urgent surgery (< 24 h) (%)	29 (67)	37 (76)	37 (60)	40 (64)	38 (58)	43(63)	41 (63)	46 (70)	37 (73)	30 (76)	0.39
ISS	30 (26–43)	29 (22–38)	29 (19–34)	29 (23–38)	29 (22–34)	27 (22–35)	29 (23–38)	29 (22–38)	34 (26–41)	32 (27–45)	0.03*
AIS head	3 (0–4)	3 (0–4)	3 (2–4)	3 (2–4)	3 (1–4)	3 (0–4)	3 (1–4)	3 (0–4)	3 (2–5)	3 (0–5)	0.003*
AIS face	0 (0–2)	0 (0–1)	0 (0–1)	0 (0–1)	0 (0–2)	0 (0–1)	1 (0–2)	0 (0–2)	0 (0–2)	0 (0–2)	0.10
AIS chest	4 (3–4)	3 (2–3)	3 (2–3)	3 (2–4)	3 (2–4)	3 (2–4)	3 (2–4)	3 (2–4)	3 (2–4)	3 (3–4)	0.008*
AIS abdomen	0 (0–3)	2 (0–3)	0 (0–2)	2 (0–3)	2 (0–3)	2 (0–3)	2 (0–3)	0 (0–3)	2 (0–2)	2 (0–3)	0.30
AIS extr/pelvis	2 (0–3)	3 (2–3)	2 (0–3)	3 (0–3)	2 (2–3)	2 (0–3)	2 (0–3)	2 (0–3)	2 (1–3)	2 (2–3)	< 0.001*
AIS external	0 (0–1)	1 (0–1)	1 (0–1)	0 (0–1)	0 (0–1)	1 (0–1)	0 (0–1)	1 (0–1)	1 (0–1)	1 (0–1)	< 0.001*
SBP_ED (mmHg)	114 (95–133)	115 (93–135)	120 (97–143)	120 (90–135)	120 (100–135)	120 (100–142)	126 (105–154)	121 (80–140)	120 (90–143)	111 (86–145)	0.58
SBP_ED < 90 mmHg (%)	8 (19)	12 (22)	12 (19)	18 (23)	14 (18)	13 (18)	9 (12)	20 (30)	15 (29)	7 (13)	0.26
Hb_ED (mmol/L)	8.2 (7.3–9.0)	8.0 (7.0–8.7)	7.8 (7.2–8.9)	8.2 (7.2–9.1)	8.0 (7.2–8.9)	8.0 (7.2–8.7)	8.3 (7.6–9.1)	8.3 (7.6–8.9)	8.2 (7.6–8.7)	8.3 (7.4–9.1)	0.38
Platelets_ED (10^9^/L)	246 (204–299)	231 (179–278)	246 (191–293)	221 (192–280)	231 (192–261)	242 (181–2610	240 (194–301)	231 (200–274)	216 (186–286)	224 (172–259)	0.53
pH_ED	7.33 (7.26–7.37)	7.30 (7.21–7.36)	7.30 (7.26–7.36)	7.32 (7.24–7.37)	7.31 (7.26–7.36)	7.31 (7.22–7.37)	7.31 (7.25–7.36)	7.30 (7.23–7.37)	7.30 (7.23–7.36)	7.29 (7.21–7.35)	0.95
PCO2_ED (mmHg)	44 (40–50)	45 (42–54)	47 (43–51)	45 (41–54)	48 (42–53)	47 (42–56)	46 (41–52)	43 (38–53)	46 (40–55)	47 (41–53)	0.58
PO2_ED (mmHg)	197 (106–277)	166 (92–279)	228 (107–307)	204 (92–315)	220 (90–325)	232 (130–344)	216 (110–375)	202 (118–310)	222 (112–347)	209 (89–331)	0.73
BD_ED (mEQ/L)	− 3.0 (− 6.5 to 0.0)	− 4.0 (− 8.0 to 1.0)	− 2.8 (− 6.0 to 1.0)	− 3.0 (− 7.0 to 0.0)	− 3.0 (− 7.0 to 1.0)	− 3.0 (− 6.0 to 0)	− 3.0 (− 5.0 to 1.0)	− 4.0 (− 8.0 to 1.0)	− 4.0 (− 7.0 to 2.0)	-5.0 (-8.0—1.0)	0.53
PT_ED (sec)	16.3 (14.7–17.9)	16.8 (15.1–19.1)	15.8 (14.4–18.0)	15.5 (14.5–18.5)	14.5 (13.3–15.7)	12.7 (12.1–13.5)	13.0 (12.0–13.7)	13.0 (12.1–13.9)	12.9 (11.6–14.1)	12.7 ( 11.7–14.4)	< 0.001*
Hb_ICU (mmol/L)	7.2 (6.6–7.9)	7.3 (6.6–7.8)	7.4 (6.6–8.3)	7.8 (6.8–8.4)	7.8 (7.0–8.4)	7.7 (7.0–8.3)	7.9 (7.1–8.7)	7.9 (7.1–8.7)	7.6 (7.1–8.2)	8.0 (7.2–8.7)	0.008*
pH_ICU	7.32 (7.29–7.39)	7.35 (7.32–7.39)	7.34 (7.28–7.39)	7.32 (7.28–7.36)	7.33 (7.27–7.37)	7.32 (7.27–7.38)	7.34 (7.28–7.37)	7.34 (7.28–7.38)	7.33 (7.28–7.38)	7.32 (7.26–7.37)	0.35
PaCO2_ICU (mmHg)	42 (39–49)	42 (37–47)	43 (37–47)	45 (40–49)	42 (38–46)	41 (36–46)	42 (36–47)	41 (37–43)	42 (35–46)	42 (40–51)	0.18
PaO2_ICU (mmHg)	160 (117–189)	148 (110–202)	141 (95–193)	142 (108–187)	158 (121–190)	143 (103–205)	165 (130–201)	144 (101–185)	161 (103–209)	145 (103–198)	0.49
BD_ICU (mEQ/L)	-2.7 (-5.3 to 1.0)	-3.5 (-5.4 to 0.6)	-3.2 (-5.3 to 1.9)	-3.6 (-6.4 to 1.5)	-4.5 (-6.9 to 2.1)	-4.6 (-6.8 to 2.3)	-4.4 (-6.9 to 2.3)	-4.4 (-6.7 to 2.8)	-4.5 ( -6.7 to 2.8)	-4.5 (-6.7 to 2.8)	0.004*
HCO3- (mmol/L)	22.6 (20.7–24.5)	21.7 (20.6–24.2)	22.3 (21.0–23.6)	22.7 (19.7–24.2)	21.3 (19.5–23.1)	21.6 (19.6–23.0)	21.8 (19.8–23.0)	20.9(19.0–22.6)	21.0(19.5–22.6)	21.4 (20.1–23.1)	0.001*

Data are expressed as median(IQR) or absolute numbers (%)

*MOI* mechanism of injury, *ISS* injury severity score, *AIS* abbreviated injury scale, *SBP* systolic blood pressure, *ED* emergency department, *Hb* hemoglobin, *PaO2* partial pressure of oxygen, *PaCO2* partial pressure of carbon dioxide, *BD* base deficit, *PT* = prothrombin time, *HCO3*^−^ bicarbonate, *ICU* intensive care unit

^*^Statistically significant

#### Resuscitation

**Fig. 2:**
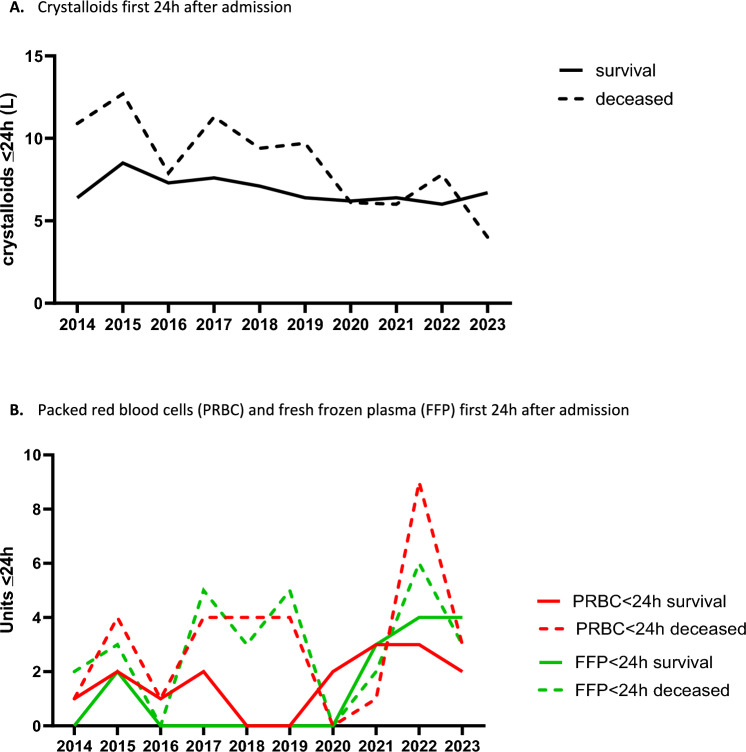
Resuscitation related to mortality in time. **A** Crystalloids first 24 h after admission. **B** Packed red blood cells (PRBC) and fresh frozen pl. (FFP) first 24 h after admission. Data are expressed in median numbers

Overall, during the 10-year time period there was a decrease in crystalloid administration (Table 3). PRBC administration in ED was higher in the later years, whereas PRBC_OR was higher in the first studied years. There was however no difference in overall PRBC ≤ 8 h and ≤ 24 h. In contrast, there was an increase in FFP in ED, and both ≤ 8 and ≤ 24 h in the later years (Table 3, Fig. 2). Interestingly, when analyzing surviving and deceased patients separately, the decrease in crystalloids was mainly caused by a decrease in patients who later died. The increase in FFP over the years was mainly seen in surviving patients (Fig. 2). There was no difference in the percentage of patients who received massive transfusion (PRBC ≥ 10 U ≤ 24 h) nor in tranexamic acid (TXA) administration (Table 3).

**Table 3:** Resuscitation per studied year

	2014 (n = 45)	2015 (n = 49)	2016 (n = 64)	2017 (n = 64)	2018 (n = 64)	2019 (n = 68)	2020 (n = 68)	2021 (n = 64)	2022 (n = 50)	2023 (n = 42)	P-Value
Crystalloids_prehosp(L)	1.0 (0.8–2.0)	0.5 (0.5–1.0)	1.0 (0.5–1.0)	1.0 (0.5–2.0)	1.0 (0.7–2.0)	0.5 (0.5–1.5)	0.5 (0.4–1.3)	1.0 (0–1.0)	0.5 (0.4–1.0)	1.0 (03–1.3)	0.02*
PRBC_prehosp (U)^#^							0 (0–0)	0 (0–0)	0 (0–1)	0 (0–0)	0.36
FFP_prehosp (U)^#^							0 (0–0)	0 (0–0)	0 (0–0)	0 (0–0)	0.79
Crystalloids_ED (L)	1.5 (1.0–1.8)	1.5 (1.0–2.0)	1.0 (0.5–1.5)	1.0 (1.0–2.0)	0.5 (0–1.5)	0.5 (0–1.5)	1.0 (0–1.0)	0.3 (0–1.4)	0.5 (0–1.0)	0.3 (0–1.0)	< 0.001*
PRBC_ED (U)	0 (0–0)	0 (0–1)	0 (0–2)	0 (0–3)	0 (0–2)	0 (0–2)	0 (0–1)	0 (0–2)	0 (0–2)	2 (0–3)	0.02*
FFP_ED (U)	0 (0–0)	0 (0–0)	0 (0–0)	0 (0–0)	0 (0–0)	0 (0–0)	0 (0–0)	0 (0–1)	0 (0–1)	0 (0–2)	< 0.001*
PLT_ED (U)	0 (0–0)	0 (0–0)	0 (0–0)	0 (0–0)	0 (0–0)	0 (0–0)	0 (0–0)	0 (0–0)	0 (0–0)	0 (0–0)	0.15
Crystalloids _OR (L)	3.0 (2.0–4.8)	3.0 (2.0–5.0)	3.0 (2.5–4.5)	2.0 (0–4.5)	1.8 (0–3.5)	2.5 (0–4.0)	2.0 (0–3.8)	2.0 (0–3.8)	2.0 (0.5–3.0)	2.5 (0–4.1)	< 0.001*
PRBC_OR (U)	2 (0–5)	2 (0–7)	1 (0–4)	0 (0–4)	0 (0–3)	0 (0–2)	0 (0–2)	0 (0–4)	0 (0–5)	0 (0–1)	0.05*
FFP_OR (U)	2 (0–4)	2 (0–5)	0 (0–2)	0 (0–3)	0 (0–3)	0 (0–3)	0 (0–4)	2 (0–5)	2 (0–5)	1 (0–2)	0.07
PLT_OR (U)	0 (0–1)	0 (0–2)	0 (0–1)	0 (0–1)	0 (0–0)	0 (0–0)	0 (0–0)	0 (0–1)	0 (0–1)	0 (0–1)	0.48
Crystalloids < 8 h (L)	4.6 (1.9–5.5)	5.5 (2.6–7.3)	4.5 (2.3–6.5)	4.9 (2.7–7.2)	4.1 (2.3–5.8)	4.9 (2.1–6.5)	3.5 (1.7–5.8)	4.0 (2.3–5.3)	4.1 (2.5–5.2)	3.9 (2.1–5.7)	0.08
PRBC < 8 h (U)	1 (0–3)	2 (0–6)	0 (0–4)	2 (0–6)	1 (0–4)	2 (0–5)	1 (0–4)	3 (0–6)	4 (0–7)	2 (0–6)	0.15
FFP < 8 h (U)	0 (0–3)	2 (0–5)	0 (0–4)	1 (0–5)	0 (0–4)	0 (0–4)	0 (0–4)	2 (0–6)	4 (0–7)	2 (0–6)	0.001*
PLT < 8 h (U)	0 (0–0)	0 (0–1)	0 (0–1)	0 (0–1)	0 (0–1)	0 (0–1)	0 (0–0)	0 (0–1)	0 (0–1)	0 (0–1)	0.05*
Crystalloids ≤ 24 h (L)	7.2 (4.4–10.3)	8.7 (6.2–11.3)	7.4 (4.9–10.5)	8.0 (5.7–11.1)	7.2 (4.6–8.9)	6.8 (4.6–10.0)	6.2 (3.5–8.9)	6.3 (4.5–8.3)	6.3 (4.5–84)	6.7 (4.2–9.3)	0.008*
PRBC ≤ 24 h (U)	1 (0–5)	2 (0–7)	1 (0–5)	2 (0–6)	1 (0–5)	1 (0–5)	2 (0–4)	3 (0–7)	4 (0–8)	2 (0–7)	0.21
FFP ≤ 24 h (U)	0 (0–4)	2 (0–6)	0 (0–5)	1 (0–5)	0 (0–4)	0 (0–6)	0 (0–6)	3 (0–9)	5 (1–10)	4 (0–9)	< 0.001*
PLT ≤ 24 h (U)	0 (0–1)	0 (0–1)	0 (0–1)	0 (0–1)	0 (0–1)	0 (0–1)	0 (0–0)	0 (0–1)	1 (0–1)	0 (0–1)	0.24
TXA ≤ 24 h (%)	23 (51)	31 (63)	44 (69)	37 (58)	39 (61)	45 (66)	47 (69)	44(69)	35 (70)	28 (67)	0.35
PRBC ≥ 10 U ≤ 24 h (%)	4 (9)	9 (18)	8 (13)	6 (9)	5 (8)	8 (12)	8 (12)	10 (16)	9 (18)	5 (12)	0.74

Data are expressed as median(IQR) or absolute numbers (%)

*Prehosp* prehospital, *ED* emergency department, *PRBC* packed red blood cells, *FFP* fresh frozen plasma, *PLT* platelets, *OR* operating room, *TXA* tranexamic acid, *U* units

^*^Statistically significant

^#^Prehospital blood product administration started from 2020

#### Outcome

**Fig. 3:**
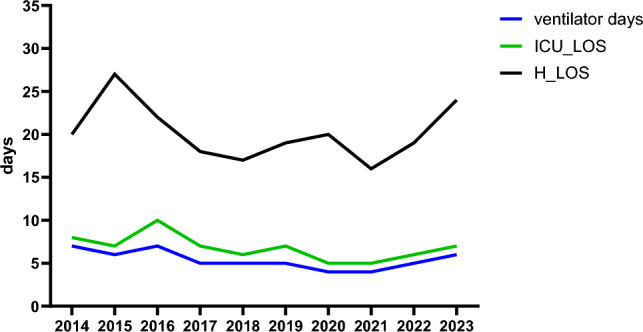
ventilator days, ICU_LOS, and H_LOS in time. Data are expressed in median numbers. *ICU_LOS* intensive care length of stay, *H_LOS* hospital length of stay

There was no difference in the number of patients who developed MODS over the years (p = 0.19), whereas ARDS became virtually non-existent over the years (p < 0.001, Table 4). Patients were shorter on the ventilator in the years 2020 and 2021.There was no significant difference in length of stay in ICU (p = 0.08) nor in hospital (0.20, Table 4, Fig. 3).

**Table 4:** Outcome per studied year

	2014 (n = 45)	2015 (n = 49)	2016 (n = 64)	2017 (n = 64)	2018 (n = 64)	2019 (n = 68)	2020 (n = 68)	2021 (n = 64)	2022 (n = 50)	2023 (n = 42)	P-value
Ventilator days	7 (3–14)	6 (2–12)	7 (3–12)	5 (2–10)	5 (2–10)	5 (2–9)	4 (2–10)	4 (2–7)	5 (2–11)	6 (3–17)	0.04*
Ventilator free days	10 (5–22)	20 (12–28)	13(6–22)	14(2–23)	12 (1–22)	13(4–21)	14 (7–21)	12 (5–22)	9 (1–25)	14 (6–28)	0.27
ICU_LOS (days)	8 (3–15)	7 (3–14)	10 (4–14)	7 (2–12)	6 (2–12)	7 (3–13)	5 (3–12)	5 (2–9)	6 (3–11)	7 (4–18)	0.08
Hospital LOS (days)	20 (11–36)	27 (15–39)	22 (13–31)	18 (9–33)	17 (10–25)	19 (9–29)	20 (12–31)	16 (10–31)	19 (10–35)	24 (9–41)	0.20
ARDS (%)	7 (16)	2 (4)	3 (5)	2 (3)	1 (2)	1(2)	0	1(2)	0	0	< 0.001*
MODS (%)	9 (20)	2 (4)	12(19)	14 (22)	10 (16)	9 (13)	10 (15)	7 (11)	11 (22)	10 (24)	0.19
Overall mortality (%)	8 (18)	7 (14)	12 (19)	15 (23)	12 (19)	14 (21)	11 (16)	10 (16)	9 (18)	8 (19)	0.98
Early death (≤ 48 h)	2 (4)	3 (6)	2 (3)	8 (13)	4 (6)	3 (4)	2 (3)	2 (3)	2 (4)	3 (7)	0.75
Late death (> 48 h)	6 (13)	4 (8)	10 (16)	7 (11)	8 (13)	11 (16)	9 (13)	8 (13)	7 (14)	5 (12)	0.65

Data are expressed as median(IQR) or absolute numbers (%)

*ICU* intensive care unit, *LOS* length of stay, *MODS* multiple organ dysfunction syndrome, *ARDS* adult respiratory distress syndrome

^*^Statistically significant

**Fig. 4:**
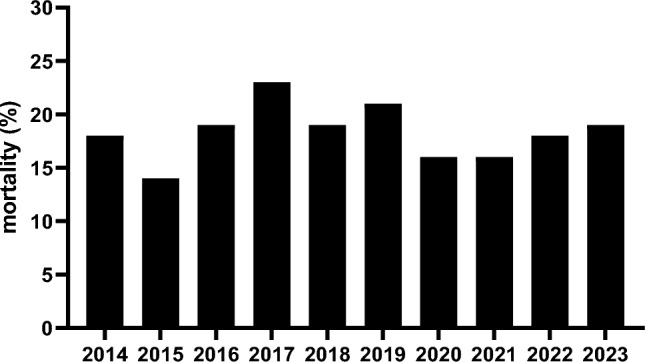
Mortality (%) per studied year

**Fig. 5:**
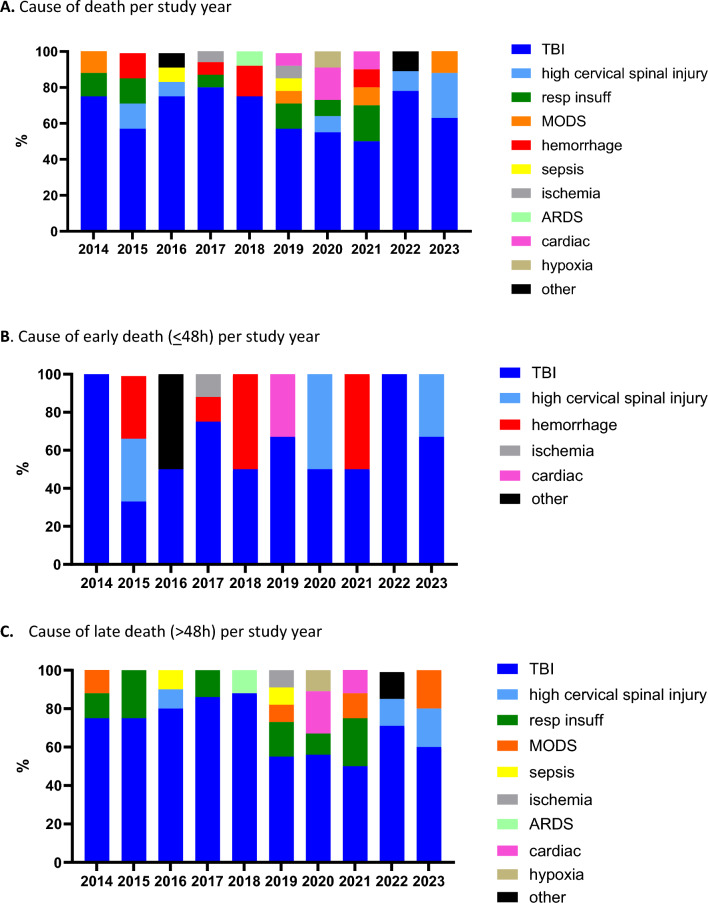
Cause of death in the studied period. **A** Cause of death per study year. **B** Cause of early death (≤ 48 h) per study year. **C** Cause of late death (> 48 h) per study year. “Other” cause of death is patient treatment was withdrawn because of the combination of injury severity and old age. *TBI* traumatic brain injury, *resp insuff* respiratory insufficiency, *MODS* multiple organ dysfunction syndrome, *ARDS* adult respiratory distress syndrome

There was no difference in mortality rate over the past 10 years, nor in the distribution of early (≤ 48 h) and late (≥ 48 h) deaths (Table 4, Fig. 4). TBI was the most common cause of death in both early and late deaths (Fig. 5) in all studied years. There was no difference in cause of death (both early and late) during the studied period (Fig. 5BC), nor was there any difference in median time of death between the studied years (p = 0.51).

## Discussion

In this study of severely injured patients admitted to the ICU, the overall mortality rate was 18%, with a preventable death rate of 0.9%. Over the 10-year study period, there was no significant change in the mortality rate, nor was there a significant difference in the causes of death. Overall, 12% of patients in the studied population died of TBI which made TBI the main cause of both early and late death in all studied years. It should be noted that in the whole polytrauma population the median AIS_head was 3 (1–4) which means a serious, but often not life-threatening injury. However, in patients who later died median AIS_head was 4 (3–5), and in patients who died of TBI median AIS_head was 4 (4–5). The high frequency of TBI related deaths is comparable to data from other studies worldwide [2, 6–8, 21, 22]. The overall death rate by exsanguination was 0.9% (5/578) in this severely injured patient population during the studied decade. This is much lower than reported in literature, even in comparable study populations [2, 6–8, 21]. Although MODS occurred in 16% of patients, MODS related mortality was 0.7% in the whole population. This was lower than several other studies [6, 8, 21–23]. The incidence of ARDS was already low in early years and seemed to be virtually non-existent anymore in later years with an overall death rate of 0.2% (one patient died of ARDS in 10 years). Likely, a combination of short transport times, short times to treatment, decrease in crystalloids, hemostatic resuscitation, damage control surgery combined with early appropriate care for fractures and lung protective ventilation has attributed to this reduction in ARDS [4, 24, 25].

Independent predictors for mortality in the whole studied population were age, AIS_head, BD_ED, and BD_ICU, PaO2_ICU and crystalloids ≤ 24 h. Since age could not be influenced, and there is not much to be done to ameliorate the effects of primary TBI, efforts should be made to optimize brain perfusion (with focus on optimizing gas exchange as early as possible and limited crystalloid administration), and minimize secondary brain injury; Abnormalities in both partial pressure of oxygen (PaO2) and partial pressure of carbon dioxide (PaCO2) are detrimental for the brain. Further, hypotension should be prevented in patients with associated TBI, and the interval between injury and definitive bleeding control is most critical to prevent worsening of the brain injury [26]. Large volumes of fluids should be avoided since they cause an imbalance in intracellular and extracellular osmolarity which on its turn ignites an inflammatory response. Inflammatory complications such as MODS and ARDS are known to be associated with large crystalloid-based resuscitation strategies [27–29]. In the last years, research on TBI has focused on targeting the pathophysiology and decreasing the inflammatory response to diminish tissue damage and cell necrosis to slow down the process of nerve degeneration [30, 31].

In this study there was no difference in age during the studied period. This is in contrast with reports suggesting that age is rising in trauma patients [7, 8, 10, 32]. This might be attributed to the inclusion criteria since patients with isolated TBI and geriatric patients with low energy injuries were excluded from this study. With an aging general population TBI will only increase and become the next epidemic in trauma. TBI constitutes a major health and socioeconomic problem throughout the world. It has not only become the largest cause of death, but survivors from severe TBI will frequently need long and intensive rehabilitation, have often a life-long disability with frequently need of assistance with activities of daily living.

ISS was different between the studied years with a rise in the later years, especially in deceased patients. This suggests that at a certain injury level death becomes almost inevitable (6/8 deaths in 2023 had an ISS > 50, 3 of them had an ISS of 75). This increase in ISS is likely even higher since AIS coding was changed from AIS98 to AIS08 in the Dutch Trauma Registry in 2015, resulting in a general decrease in ISS in later years [33].

It is worth mentioning that patients included in this study did not exhibit severely disrupted physiology upon arrival in ED despite severe injuries (ISS 29) and 65% of them requiring urgent surgery. This occurrence, where severely injured patients from smaller service areas with brief transport times do not display disrupted physiological parameters upon arrival has been documented before [24].

It remains unclear why Prothrombin time (PT) measured in ED was prolonged in the first years since all other physiological and demographic parameters in ED were similar over the years. We did not collect data on anticoagulant use, but there is no reason to suspect that anticoagulant use has decreased over the years. It is however possible that this decrease in PT was influenced by the type of anticoagulant used, since there has been an overall change from vitamin K antagonists to non-vitamin K antagonist oral anticoagulants (NOAC) use which has minimal effect on PT [34].

Hemoglobin in ICU increased likely due to early PRBC administration both prehospitally and in ED. First measured BD and bicarbonate in ICU decreased even though pH remained unchanged. We have no clear explanation for this phenomenon since other parameters that are an expression of physiologic derangement were unchanged.

Although generally still a fairly large amount of crystalloids ≤ 24 h was given, a decrease in the usage of crystalloids was observed in ED, OR and first 24 h during the studied period. Interestingly, crystalloid reduction was most obvious in patients who later died. This could be explained by an increased reluctance to overload polytrauma patients with associated TBI with fluids. Likely, the implementation of initial administration of isotonic fluids from 2 to 1 L as recommended in the 10th edition in the ATLS Advanced Trauma Life Support in 2018 has attributed to an overall decrease in crystalloid administration [35]. The decrease in early crystalloid administration has probably influenced blood product administration since there was an increase in administration of PRBC and FFP in ED. FFPs were also increasingly administered ≤ 8 and ≤ 24 h. The increase in FFPs was most apparent in patients who survived suggesting that FFP resuscitation in these patients was beneficial.

Over the years there was a decrease in ventilator days, this was most obvious in 2020 and 2021. Also, ICU_LOS was lowest in these 2 study years. There was however no difference in mortality rate nor in time to death in those years compared to other years. Likely, these lower ventilator days and ICU_LOS were influenced by the COVID era, when ICU beds were limited [36].

As far as we know, this is the first paper to systematically observe a series of consecutively severely injured patients over a span of ten years, offering comprehensive data on their physiological status, early interventions, and eventual outcomes for comparison. One limitation was that the study was conducted at a single center, where both research and clinical care were overseen by the same team of practitioners. Additionally, no information regarding pre-existing conditions was recorded, and in only half the patients Glasgow Coma Scale scores were collected. Furthermore, data wasn't collected for patients who arrived deceased at the ED or those who passed away before being admitted to the ICU. Despite these limitations, we believe that the cohort studied, predominantly comprising cases of blunt trauma in a densely populated area with short transportation times, can be seen as representative of similar urban settings with a similar prevalence of blunt trauma incidents.

In conclusion, in this 10-year analysis of severely injured patients admitted to ICU there was no difference in mortality rate despite an increase in ISS in the later years. TBI was the single largest cause of both early and late death in all years. Demographics and early physiology were remarkable similar. Hypoxia and base deficit in ICU, and crystalloid resuscitation were independent predictors for mortality that could be influenced to potentially decrease secondary brain damage. Less crystalloids and more blood products in the first 24 h were administered over the years. It will be challenging to further improve physiology to diminish TBI related deaths while maintaining the current level of care. With an increasing elderly population, TBI will become the next epidemic in trauma in the near future. Research should not only focus on (primary and secondary) prevention of brain damage, but also on decreasing the inflammatory response as was successfully done in other parts of the body in the past decades.

## Supplementary Information

Below is the link to the electronic supplementary material.

Supplementary file1 (DOCX 23 KB)

Supplementary file2 (DOCX 14 KB)

## Data Availability

The dataset supporting the conclusions of this article can be obtained upon reasonable request from the corresponding author.
