# Supplementary material for: TBI related death has become the new epidemic in polytrauma: a 10-year prospective cohort analysis in severely injured patients
Source: Eur J Trauma Emerg Surg. 2024 Sep 17;50(6):3083–94. doi: 10.1007/s00068-024-02653-1 (PMC11666694; doi:10.1007/s00068-024-02653-1)
Supplement: Supplementary file 1 — Supplementary file1 (DOCX 23 KB) [file 68_2024_2653_MOESM1_ESM.docx]

**Figure S1.** Flowchart of included patients

**Total number of trauma patients (age>15) admitted to hospital 2014-2023**

n=10596

**Patients admitted to ICU, directly or via OR**

n = 1845

**Isolated TBI* (including asphyxiation, drowning, burns)**

n =1267

**Severely injured patients admitted to ICU**

n = 578

* Isolated traumatic brain injury (TBI) was defined as Abbreviated Injury Score (AIS) head >3 and AIS <2 or less in other regions
