# Supplementary material for: TBI related death has become the new epidemic in polytrauma: a 10-year prospective cohort analysis in severely injured patients
Source: Eur J Trauma Emerg Surg. 2024 Sep 17;50(6):3083–94. doi: 10.1007/s00068-024-02653-1 (PMC11666694; doi:10.1007/s00068-024-02653-1)
Supplement: Supplementary file 2 — Supplementary file2 (DOCX 14 KB) [file 68_2024_2653_MOESM2_ESM.docx]

**Table S1.** Type of traumatic brain injury in patients with AIS_head>3 (n=346)

| Type of brain injury | Nr of injuries* |
| --- | --- |
| Brain stem contusion | 3 |
| Brain stem infarction | 1 |
| Brain stem hemorrhage | 4 |
| Brain ischemia | 1 |
| Brain swelling/oedema | 105 |
| Contusion | 81 |
| Diffuse axonal injury | 30 |
| Epidural hematoma | 43 |
| Intracerebral hematoma | 46 |
| Intraventricular hemorrhage | 28 |
| Subarachnoid hemorrhage | 93 |
| Skull base fracture | 202 |
| Skull vault fracture | 31 |
| Subdural hematoma | 133 |
| Total | 801 |

*Several patients had more than one type of injury
